# The symptom experience of hereditary angioedema (HAE) patients beyond HAE attacks: literature review and clinician interviews

**DOI:** 10.1186/s13023-022-02360-3

**Published:** 2022-06-16

**Authors:** Milenka Jean-Baptiste, Robbin Itzler, Subhransu Prusty, Dylan Supina, Mona L. Martin

**Affiliations:** 1 Evidera, Bethesda, MD USA; 2 CSL Behring, King of Prussia, PA USA; 3 CSL Behring GmbH, Marburg, Germany; 4 Jazz Pharmaceuticals (Formerly CSL Behring), King of Prussia, PA USA; 5 Evidera, 615 2nd Ave Ste 500, Seattle, WA 98104 USA

**Keywords:** Hereditary angioedema, HAE symptoms and impacts, C1-INH, HRQoL, Between attack symptoms

## Abstract

**Background:**

Hereditary angioedema (HAE) is a genetic disorder characterized by re-occurring swelling episodes called “attacks,” usually in the limbs, face, airways, and intestinal tract. New prophylactic therapies have reduced the frequency of these attacks. This study describes results from a literature review and clinician interviews assessing patient HAE symptom experiences and timing, and then evaluates whether existing patient-reported outcome (PRO) tools adequately reflect this experience.

**Methods:**

A targeted literature review as well as interviews with key opinion leaders (KOLs), were conducted to capture information about the patient experience and their symptoms. An assessment of various PROs was then conducted to determine how well they each covered HAE symptoms and impacts.

**Results:**

Nineteen HAE symptoms were identified. KOLs reported that patients on prophylactic therapy experienced some symptoms indicating an attack was imminent, but then never experienced an attack. The comparison of the different PROs found that the Hereditary Angioedema Patient-Reported Outcome was the instrument that most thoroughly examined the symptoms of patients with HAE.

**Conclusions:**

Given the introduction of new prophylactic therapies, further research is needed to determine the effect of being attack-free for longer periods of time on health-related quality of life.

## Introduction

Hereditary angioedema (HAE) is a genetic disorder characterized by re-occurring swelling episodes, usually in the limbs, face, airways, and intestinal tract [1]. HAE is estimated to impact one in 50,000 people [2, 3] and can potentially be life threatening, as swelling in the airways can lead to asphyxiation [4]. HAE onset can occur at any age [5], affecting males and females equally [2]. Episode or attack frequency can vary. However, attacks have occurred as often as every one to two weeks [3], and the swelling associated with an attack can last 2–5 days [6]. HAE attack triggers can vary greatly across patients, and in some cases, are unclear. Generally, triggers include emotional factors (such as anxiety or fear), physical stressors (such as exercise), and environmental factors [4].

Angioedema in the extremities and abdomen is the most common clinical symptom of an HAE attack. These main symptoms relate to large amounts of shifting fluids, and pain related to edema around the face, joints, and extremities [1, 7]. While patients often present with a wide variety of symptoms, research to date has focused on the HAE symptoms that occur hours (most often) and days [7] before an attack, as well as during acute swelling from an HAE attack [8]. Very little information is available on symptoms experienced outside of the attack period by patients living with HAE, such as the days following the attack (when the swelling is subsiding), and the variable periods between HAE attacks.

Generally, treatment has focused on limiting morbidity and preventing mortality—thus improving health-related quality of life (HRQoL) [9]. Typical therapies include rescue medications that immediately treat attacks and prophylactic medications that prevent attacks. Different therapies types have included monoclonal antibodies, attenuated androgens, tranexamic acid, and esterase inhibitors [4, 10]. However, a considerable burden related to both HAE and its treatment still exists [10], which needs to be better understood in relation to how it impacts patients’ lives [11].

In recent years, several new, long-term, prophylactic HAE therapies have become available in various markets. Haegarda® is an example of a plasma-derived concentrate of C1-esterase inhibitor (human) (C1-INH) for subcutaneous administration twice weekly. Lanadelumab (Takhzyro®) is a plasma kallikrein inhibitor (monoclonal antibody) also used subcutaneously once every two weeks. Dosing every four weeks may be considered in some patients, according to the Food and Drug Administration (FDA) package insert [12]. Berotralstat (Orladeyo®) is an oral plasma kallikrein inhibitor administered once daily. As new prophylactic therapies are introduced, clinicians report observable changes in how their HAE patients experience symptoms—specifically, in terms of symptom type, severity, duration, pattern of occurrence, and frequency of HAE attacks. Variability in symptom experienced can be observed between patients, and in different attacks experienced by the same patient [11].

HAE symptoms across different timepoints in the HAE patient experience need to be considered and carefully defined. To date, the literature has focused on prodromal symptoms occurring just before swelling, during swelling, and immediately after the swelling subsides. However, there is limited evidence of the patient symptom experience in-between attacks. More detailed descriptions are needed, as symptoms and attacks change as patients receive treatment with the newer, long-term prophylactic therapies. Information is also needed on how these changes affect patients’ lives. In turn, this helps determine whether the most important concepts of the patient experience are being assessed.

Recent consensus reports on the burden of disease indicate that HAE may be associated with significant disease burden, which interferes with a patient’s quality of life both during and between attacks [13]. Determining HAE disease burden includes assessing attack frequency and severity, as well as quality of life effects. Changes in the patient experience raise questions about the adequacy of current assessment tools to capture symptom changes across the patient HAE experience. When used correctly and consistently, these assessment tools can improve individualized treatment plans. In doing so, they could potentially reduce a patient’s annual treatment costs [14]. Finally, these assessment tools can also determine whether existing study endpoints efficiently evaluate new therapies. In recent years, new instruments have been developed to better assess HRQoL aspects in this population, but it remains unclear whether these tools fully capture the patient experience with HAE symptoms, HAE’s overall burden, and the ability to reflect changes.

The primary aim of this paper is to describe the full range of symptoms reported by patients living with HAE and reflected in the literature. This paper also examines the content coverage of current measurement tools, to see if they are adequately capturing symptoms and impacts reported in the literature and by clinicians.

## Materials and methods

A targeted literature review was conducted to identify the common symptoms associated with HAE outside of the attack phase, and to time the symptoms in relationship to an HAE attack. These symptoms could have occurred just before an attack (also known as “prodromal symptoms”), immediately after the attack as the swelling subsides, and between attacks. Clinical expert interviews (n = 11) were then conducted to further describe the HAE symptoms discussed by patients with their doctors, the timing and duration of symptoms, and the impressions of clinicians from patient reports about symptom changes following the start of long-term prophylactic treatment. Finally, a comparison of content coverage was conducted for the existing HAE-specific assessment tools, to identify whether they were adequately addressing the patient-reported symptoms and impacts on new prophylactic therapies.

A targeted search strategy was developed to initiate the literature review. OVID and Medline databases were searched using the categories listed in Table 1. Limits were applied to include only those articles published in English between 2009 and 2019, and conducted with humans. Prior to screening the records using the eligibility criteria, duplicate entries were identified and removed. Records were then screened using the criteria described below.

**Table 1:** Search categories used in the literature review

Category	Search term used
Indication	Angioedema hereditary, hereditary angioedema
Period of time	Non-attack, attack-free, basal, prodromal, period, interval, phase, between attacks
Additional Key Words	Symptoms, prodromal symptoms

Articles were included if they focused on describing HAE patient symptoms, and described at least one symptom experienced by patients outside the swelling attack period. Reference lists from full-text articles were also reviewed to identify additional, potentially relevant citations. A data extraction template was developed to aid in the literature review. Information extracted from the literature included patient experiences with symptoms and impacts, as well as information about any assessment tools used in these studies.

US-based clinical experts were identified from those frequently presenting at HAE meetings, as well as published specialists who are active in HAE research and treatment. Nineteen clinicians were identified from academic centers, large referral centers, and private practice; eleven agreed to participate in the qualitative interviews. Reasons for non-participation included lack of time to be involved, prohibitive contracting issues, and conflict of interest. A semi-structured qualitative interview guide was developed to facilitate discussion with clinical experts regarding HAE symptoms described by patients. Clinician interviews were conducted by trained qualitative interviewers and lasted approximately 60 min. Sample interview guide questions included the following:

- What are the most common prodromal symptoms that you hear about from your patients who still have HAE attacks?
- What do your patients usually describe as the impacts on their daily lives because of these symptoms that they have before, after, and between HAE attacks?
- When you think about the symptoms that your patients have in between their HAE attacks, what are your thoughts about the source of these symptoms?
- When you think about the symptoms that your patients have in between their HAE attacks, what are your thoughts in general about treating and managing these between-attack symptoms?

Interviews were audio-recorded and transcribed; transcripts were used for qualitative analysis. Areas of key interest included patients’ descriptions of their symptoms, when those symptoms occurred relative to the swelling attack, and the types of changes reported following the start of long-term prophylactic treatment.

The resulting symptom and impact concepts identified in both the literature and clinician interviews were used to develop a conceptual disease model (CDM) that describes the patient experience with symptoms, impacts, severity, and treatment. The most frequently cited symptoms and impacts from the literature review were used to develop the CDM. This was later updated using the clinician interview results.

**Fig. 1:**
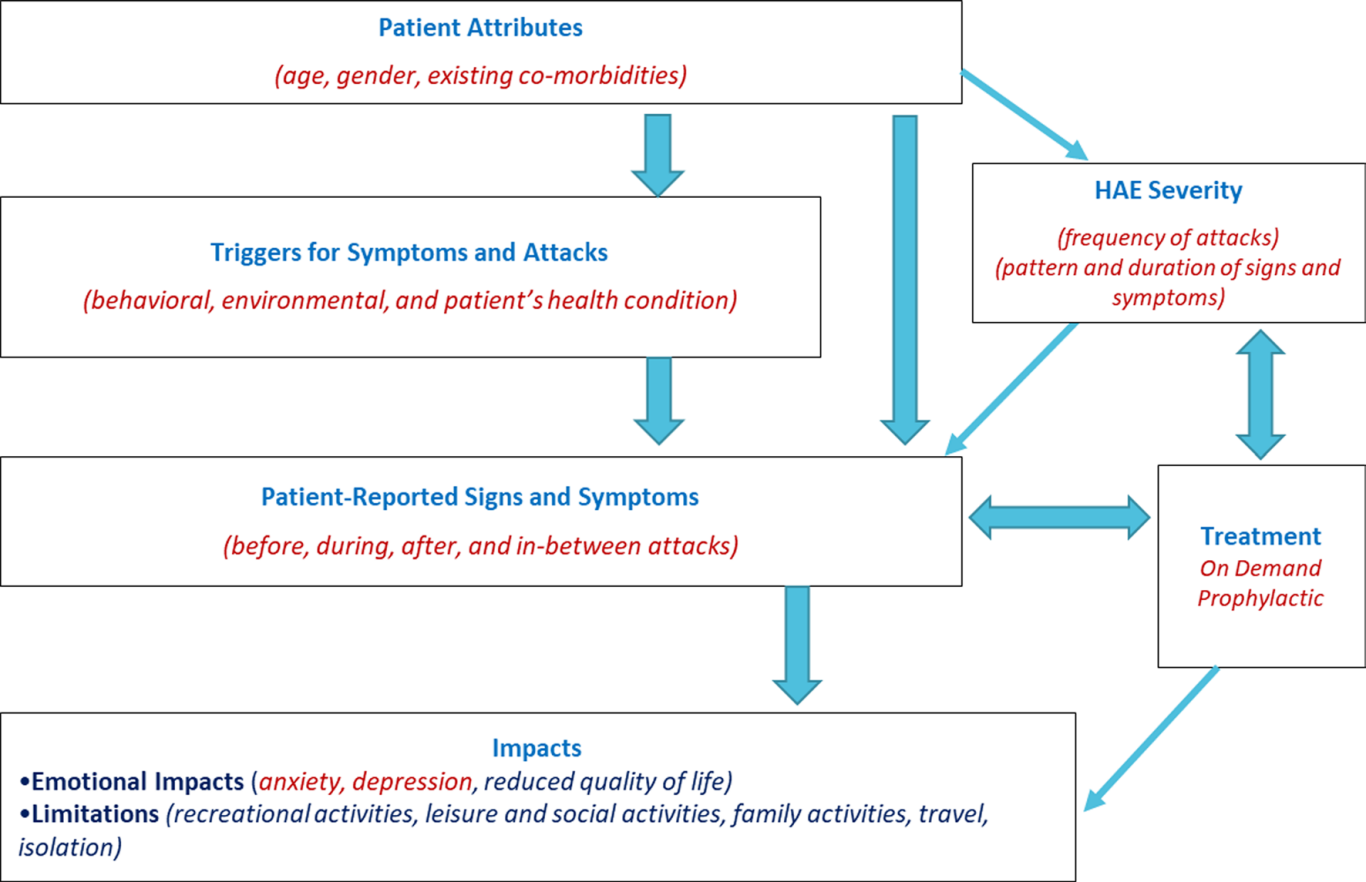
Conceptual (disease) model for HAE

While previous work went into the development of HAE-specific disease models [15], our CDM describes the patient-reported signs and symptoms before, during, after, and in between attacks. It also describes how HAE severity and the patient’s treatment choice influence one another. Reduction in HAE severity may then lessen limitations on recreational activities, social factors, and travel; as well as emotional impacts like anxiety, depression, and reduced quality of life (Fig. 1).

Several HAE-specific HRQoL assessment tools (PROs) were identified during the literature review. These PROs were reviewed for specific content coverage across the spectrum of patient-reported symptoms and impacts. The PROs that were identified as HAE-specific included the following: the 17-item Angioedema Quality of Life Score (AE-QoL) [16], the 25-item Hereditary Angioedema Quality of Life Score (HAE-QoL) [17], the recently developed 27-item Hereditary Angioedema Association Quality of Life Score (HAEA-QoL) [18], the 12-item Hereditary Angioedema Activity Score (HAE-AS) [19], the 18-item Hereditary Angioedema PRO (HAE-PRO) [20], the Angioedema Control Test (AECT) [21], and the five-item Angioedema Activity Score (AAS) [22]. A matrix was developed to map the symptoms and impacts covered in each PRO, comparing the content coverage of these measures to the spectrum of patient-reported symptoms and impacts from the published literature.

## Results

### Literature review

**Fig. 2:**
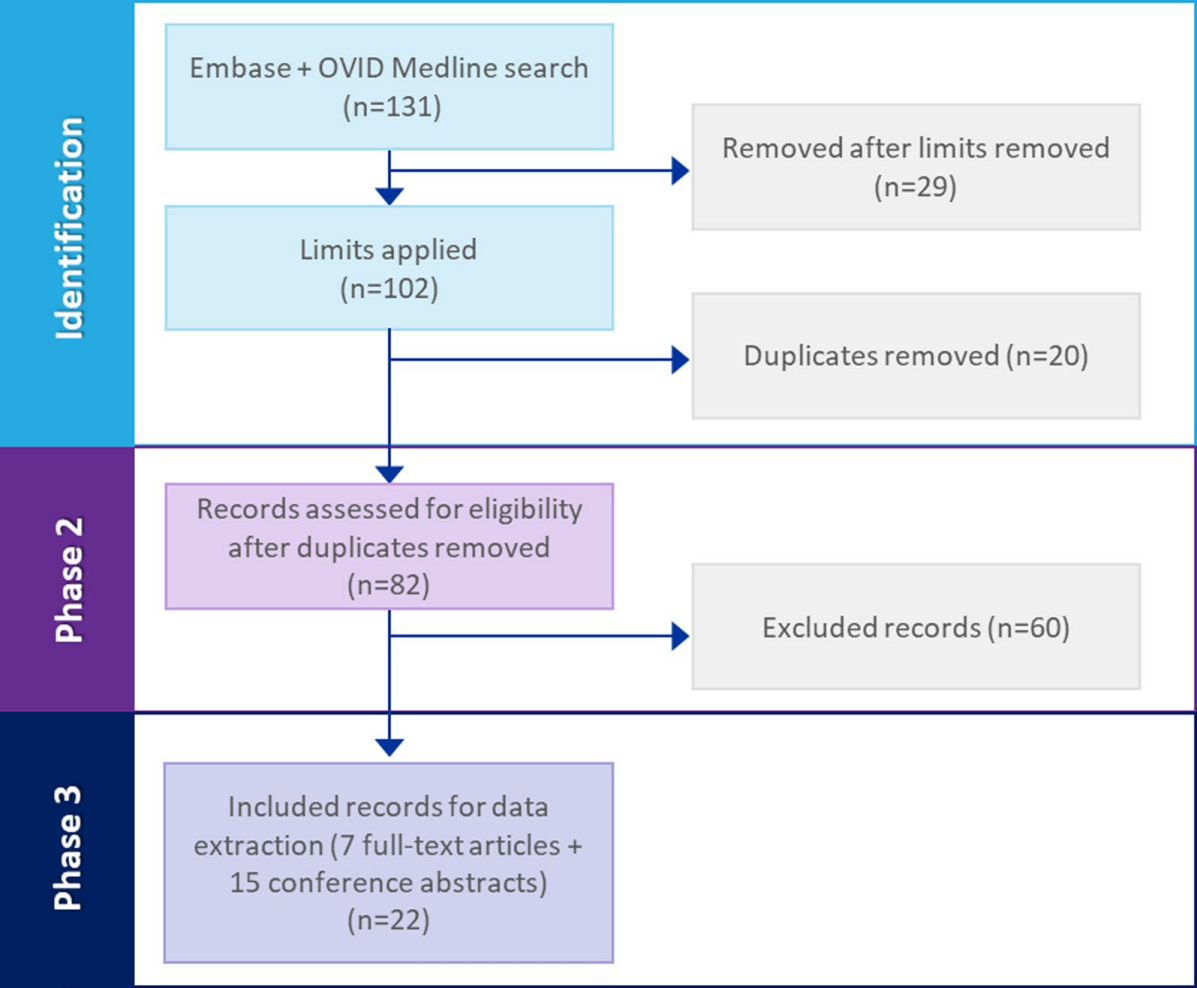
Abstracts identified, screened, and selected to review

Of the 131 records identified in the literature review, 29 were removed after limits were applied. Articles removed at this point included those published in a language other than English, outside the 2009 to 2019 timeframe, and on non-human subjects. Twenty duplicate entries were also removed, leaving 82 records to be screened using the eligibility criteria. Sixty records did not meet the eligibility criteria, which yielded seven full-text articles and 15 conference abstracts for review and data extraction. The studies covered in the published literature presented information from the patient perspective, with one exception—presenting information based on the clinician’s view of the patient perspective (Fig. 2).

After combining different terminologies used to express the same symptoms, 20 types of symptoms were extracted from the literature to represent the HAE patient experience. The most frequently reported symptoms (aside from swelling) were fatigue, erythema marginatum, paresthesia, stinging, tingling, abdominal issues (i.e., cramping, stomach ache), nausea, vomiting, pain, rash, flu-like symptoms, pruritus, and rumbling throughout different body parts. Additional symptoms reported included irritability/short temper, bruises, transitory edema, appetite changes, headache, fatigue, restlessness, excessive perspiration, changes in body temperature, and psychological issues (including depression/sadness and anxiety).

Although patient symptoms were identified throughout the literature, the timing of symptoms occurrence was most often described as “prodromal” (signaling the beginning of an attack) or during the swelling episode. Symptoms were seldom reported as occurring while the swelling subsided or in between attacks.

### Clinician interviews

Eleven interviews were conducted with the identified clinical experts. Four clinicians were from academic centers, two were from large referral treatment centers, and five were in private practice. The private practice settings treated 25–100 HAE patients, and the case load at the larger academic centers ranged from 100 to 180. The majority (75–95%) of the patient groups treated by interviewed clinicians were type I or type II HAE.

Although there was broad acknowledgement that symptoms occurred outside of the swelling episodes, there was no consensus on how to precisely define the attack period—as well as the post-attack period—versus those symptoms occurring between attacks. In describing prodromal symptoms, most clinicians (n = 8) described this as any symptom experienced within a couple hours to a couple of days before the swelling episode (or the attack), whereas a few characterized these symptoms as specifically attack-related. Most clinicians (n = 8) described patients experiencing symptoms occurring just prior to the swelling, during the actual swelling episode, after an episode as the swelling subsides, or in between episodes—which excludes the other time periods described above. This reflects how HAE symptoms could occur on a continuum.

While clinicians reported many of the same symptoms appearing in the literature, their interviews added more detail about these symptoms. For example, the physicians focused on different types of swelling based on the anatomical locations involved, and the timing of the symptoms in relation to swelling episodes. Clinician-reported symptoms included general swelling, facial swelling, throat swelling, and peripheral angioedema in the hands or feet. Other non-swelling symptoms mentioned during clinician interviews included fever, general aches and pain or muscle aches, numbness, the sensation of feeling “run down” or having fatigue, and non-swelling abdominal symptoms (cramping, loose stools, nausea, vomiting). Except for the primary swelling symptoms that characterize HAE attacks, the symptoms that patients reported to clinicians occurred both before their HAE swelling attacks, and again after the swelling attack had peaked. In some cases, fatigue and abdominal symptoms continued into the period between HAE attacks. Other symptoms like rash, flu-like symptoms (fever, chills, etc.), and various paresthesias (burning, prickling, etc.) were experienced just prior to and following the acute attacks (as the swelling subsides). Of note, these were not reported in between attacks.

Finally, emotional symptoms (sadness, depression, worry, fear, anxiety) were reported throughout the HAE patient experience. Anxiety would focus on when another attack would begin. Once the attack started, the anxiety would switch to how severe the swelling would be before subsiding. Sadness and depression were experienced throughout the different HAE phases, related to the quality-of-life disruption.

One of the most prominent changes clinicians heard from patients being treated prophylactically with C1 INH inhibitors (such as Haegarda® and Firazyr®) or monoclonal antibodies (like Takhzyro®) was that they still experienced symptoms associated with attack onset—but now that they were on prophylactic treatment, these symptoms were not followed by an attack.

### Symptom timing in relation to HAE attacks (swelling episodes)

**Fig. 3:**
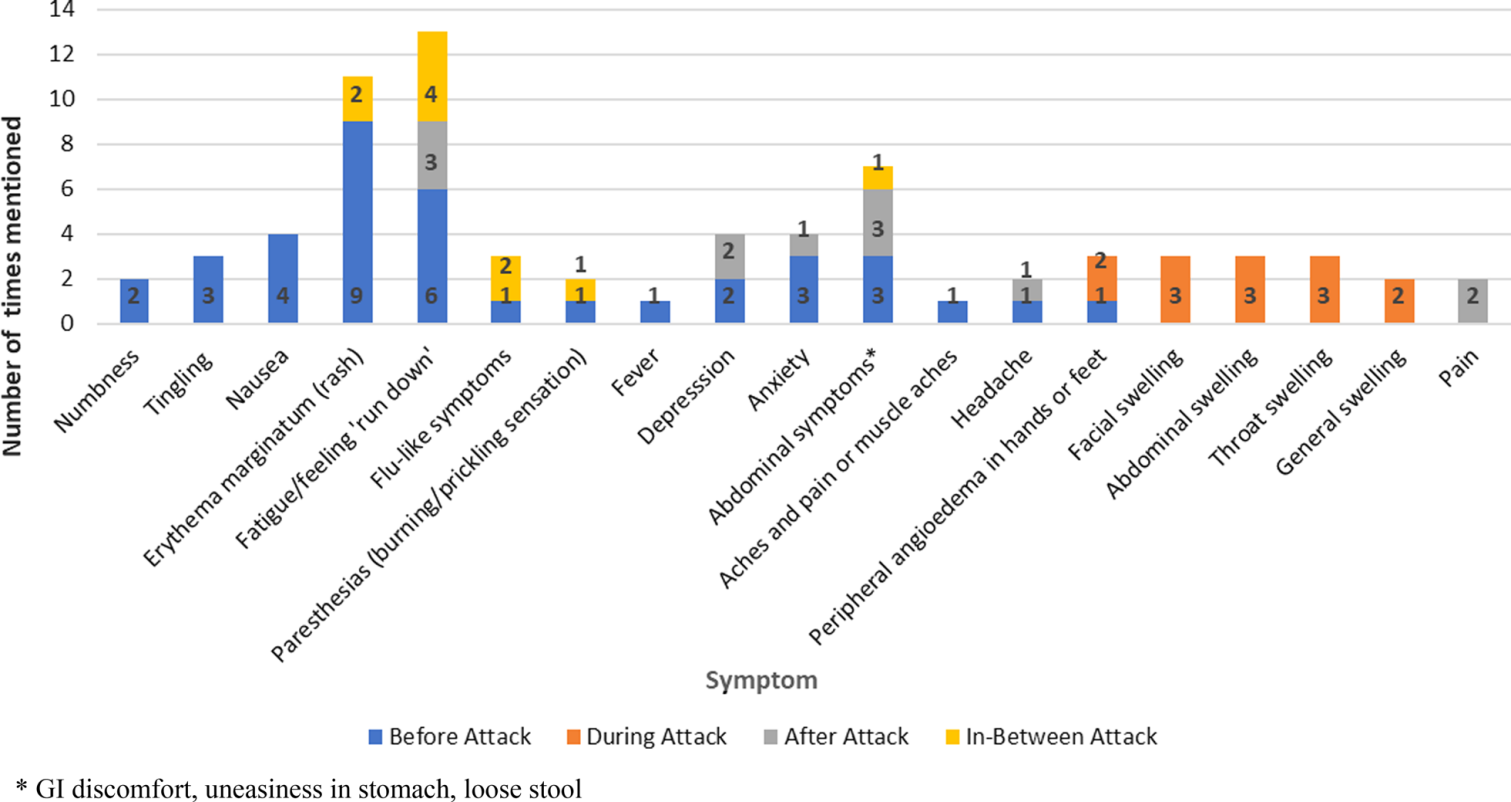
Time period where symptoms were experienced

As noted earlier, some of the symptoms reported in the literature were not always categorized by the timing in relation to swelling episodes. Thus, this information was not available for all symptoms. Also, clinicians’ responses regarding when their patients’ experienced symptoms were based on anecdotal recall of conversations with patients; it should be noted that this is not a typical line of inquiry in the clinical setting. Therefore, timing for only 19 symptoms was reported in the literature and clinician interviews (Fig. 3). Fourteen of these 19 symptoms (74%) occurred before the onset of an actual attack, five (36%) occurred during an attack, six (43%) occurred after an attack, and five (36%) occurred between attacks. Nine symptoms (64%) also occurred across multiple timeframes.

Of the 19 symptoms identified in the literature review and clinician interviews with a timing designation, rash and fatigue were most often identified before and after an attack. Clinicians described swelling in the hands, fingers, feet, face, and abdomen as the most common prodromal symptoms—as well as key attack symptoms. Rash and fatigue were also commonly cited in the literature as prodromal.

### CDM description

The CDM describes how various environmental, behavioral, and health conditions—in combination with patient attributes—influence HAE severity, swelling severity and frequency, and the pattern and duration of signs and symptoms. HAE severity influences treatment choice—particularly for long-term prophylaxis, which in turn influences the patient’s symptom experience, emotional impacts, and activity limitations (Fig. 1).

### Content analysis of existing assessment tools

Several assessment tools were mentioned across both the literature review and clinical expert interviews. Those considered for content comparison include the five-item AAS [23, 24], the four-item AECT [21, 25], the 17-item AE-QoL [16, 26], the 18-item HAE-PRO [20], the 12-item HAE-AS [19], the 25-item HAE-QoL [27], and the 27-item HAEA-QoL [28, 29].

The content of these tools was mapped to compare differences between measures. The PROs identified varied in length. Some were four-item measures, while others included up to 27 items. The recall periods also varied, ranging from “None defined” (HAEA-QoL and HAE-PRO) to “past 6 months” (HAE-QoL and HAE-AS). This is important to note because clinicians described widely varying timeframes for both symptom occurrence and intervals between attacks. No instruments inquired about the time period where symptoms occurred—effectively preventing the assessment of changes in symptom and attack timing that many patients starting prophylaxis experience. Table 2 shows the coverage of symptom concepts in existing instruments.

**Table 2:** Symptom coverage in existing HAE specific instruments

SYMPTOM—content coverage of existing HAE-specific instruments	AE-QOL 17 items	HAE PRO 18 items	AECT 4 items	AAS 5 items	HAE-AS 12 items	HAE-QOL 25 items	HAEA-QOL 27 items
Leg/foot swelling		Yes			Yes		
Arm/hand/finger swelling		Yes			Yes		
Face swelling					Yes		
Throat swelling		Yes					
Abdominal swelling		Yes			Yes		
Genital swelling					Yes		
Swelling (unspecified)				Yes			
Tiredness/ fatigue/ energy levels		Yes					Yes
Pain (muscle pain, joint pain, abdominal pain)		Yes					
Skin symptoms (pain, redness, irritation)		Yes					
Difficulty swallowing/ voice change		Yes					
Respiratory difficulty					Yes		
Headache		Yes					
Dizziness							
Other abdominal symptoms (such as cramps, loose stools)		Yes (Diarrhea Constipation)					
Nausea		Yes					
Vomiting		Yes					
Difficulty urinating		Yes					
Overall frequency of attacks			Yes				
Overall severity of attack		Yes					
General duration of symptoms		Yes					
Severity of swelling overall				Yes			
Severity of pain/ discomfort overall				Yes			
Time of day swelling took place				Yes			
Recall Period	Last 4 weeks	None defined	Past 3 months	Past 24 h	Past 6 months	Past 6 months	None defined

*AAS* Angioedema activity score, *AECT* angioedema control test, *AE-QoL* angioedema quality of life score, *HAE-PRO* hereditary angioedema PRO, *HAE-AS* hereditary angioedema activity score, *HAE-QoL* hereditary angioedema association quality of life, *HAEA-QoL* hereditary angioedema association

The AE-QoL is largely a quality of life measure more focused on impacts— not symptom coverage. Emotions such as anxiety or depression are coded with impacts, since they are a disease impact rather than a direct HAE symptom. The HAE-PRO and HAE-AS have detailed coverage of the swelling symptoms associated with attacks, but the HAE-PRO is the only instrument that provides any substantial coverage of other non-swelling symptoms. Only the HAE-PRO and HAEA-QoL address fatigue, which was frequently reported in both the literature review and clinician interviews. Only the HAE-PRO assesses attack severity and symptoms duration, and only the AAS addresses the overall severity of swelling and pain (though in general, rather than for specific swelling locations).

Interference with work, productivity, and daily activities are covered in varying detail by several instruments. The primary categories of life interference and burden identified in the literature—and mentioned in the clinician interviews—were the impact on work and productivity, social relationships, emotions, and the ability to resume greater normalcy in daily life activities. The HRQoL and impact coverage of each assessment tool are further described in Table 3.

**Table 3:** Impact coverage in existing HAE-specific instruments

IMPACT—content coverage of existing HAE specific instruments	AE-QoL 17 items	HAE-PRO 18 items	AECT 4 items	AAS 5 items	HAE-AS 12 items	HAE-QoL 25 items	HAEA-QoL 27 items
Ability to work/productivity	Yes	Yes			Yes	Yes	
Ability to do physical activity	Yes						
Ability to carry out daily activities		Yes		Yes			Yes
Leisure time	Yes						Yes
Social relationships	Yes					Yes	Yes
Eating and drinking restricted	Yes						
Sleep difficulties	Yes						
Tired during the day	Yes						
Trouble concentrating	Yes						
Burden experienced by swelling	Yes						
Ability to go to school					Yes		
Going out in public							Yes
Ability to travel						Yes	Yes
ER visit due to HAE attack					Yes		
HAE controlled by treatment			Yes				
Worry about aspects of care and treatment							Yes
Bothered from unpredictability of HAE attack			Yes				
General health					Yes	Yes	
General quality of life			Yes				
Bothered from unpredictability of HAE attack			Yes				
Emotional impacts							
Feeling of enjoying life							Yes
Mood/depression	Yes						Yes
Anxiety/fear/worry	Yes					Yes	Yes
Embarrassment/shame (appearance)	Yes					Yes	Yes
Recall period	Last 4 weeks	None defined	Past 3 weeks	Past 24 h	Past 6 months	Past 6 months	None defined

*AAS* angioedema activity score, *AECT* angioedema control test, *AE-QoL* angioedema quality of life score, *HAE-PRO* hereditary angioedema PRO, *HAE-AS* hereditary angioedema activity score, *HAE-QoL* hereditary angioedema association quality of life, *HAEA-QoL* hereditary angioedema association

While some measures focus more on assessing the patient’s symptomatic experience, others more broadly assess HAE disease burden—specifically, how much HAE might impact an individual’s daily functioning or overall quality of life. In this sense, disease burden reflects the degree to which symptoms might interfere with daily activities or overall quality of life. Although the AE-QoL, HAEA-QoL, and HAE-QoL do not cover symptoms, these two measures do have better coverage for emotional impacts. Similarly, the four-item AECT also evaluates HRQoL, specifically by assessing symptom bother and how well the prescribed therapy controls HAE. In looking across Tables 2 and 3, instruments like the HAE-PRO and the HAE-AS show both symptomatic and impact content coverage.

When describing patient-reported symptom changes, clinicians described significant changes in severity and duration with both C1 INH inhibitors and monoclonal antibodies. Only the HAE-PRO asks participants to describe symptom severity and duration. However, the AECT describes how bothersome these symptoms were and how well they are controlled by treatment.

#### Instrument content compared to literature review and clinician interview results

In addition to comparing each PRO’s content to each other, we also compared the symptoms and impacts identified in the literature and clinical expert interviews (Table 4). The 29 identified symptom concepts were grouped into similar categories and listed in the left column**.** The symptoms that are covered in existing HRQoL instruments have been highlighted in blue. The symptoms reported in the literature and discussed by clinicians but not covered in existing HAE instruments have been highlighted in green.

**Table 4:**
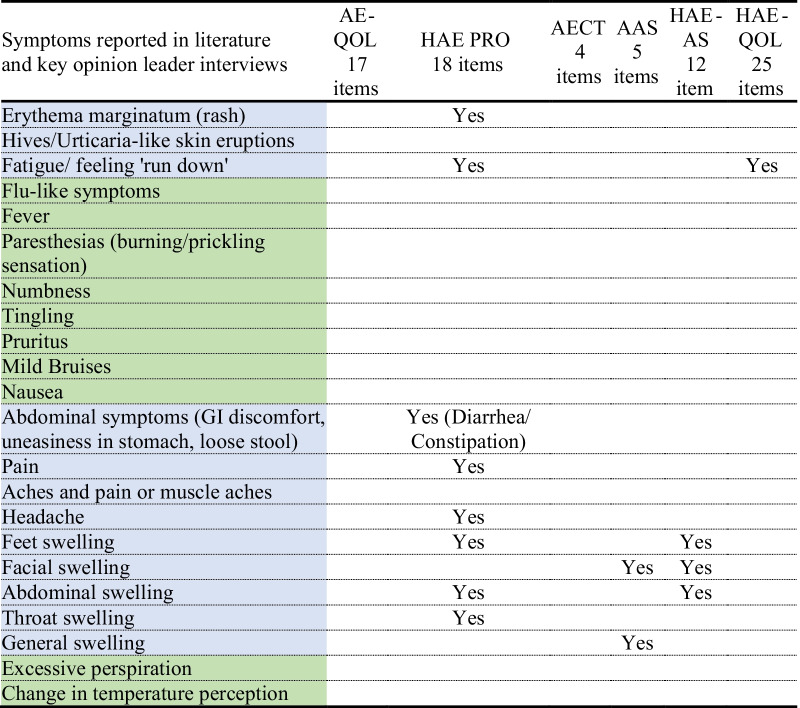
Coverage in existing HAE-specific instruments for symptoms reported in literature and by clinicians

*AAS* Angioedema activity score, *AECT* angioedema control test, *AE-QoL* angioedema quality of life score, *HAE-PRO* hereditary angioedema PRO, *HAE-AS* hereditary angioedema activity score, *HAE-QoL* hereditary angioedema association quality of life, *HAEA-QoL* hereditary angioedema association

Blue cells = Symptom identified in at least one instrument; Green cells = Symptom not included in any instrument

Based on this comparison, only the HAE-PRO, AAS, HAE-AS, and HAEA-QoL adequately assessed the symptoms described in the literature review and by clinicians. Of these four instruments, only the 18-item HAE-PRO identified more than three symptom categories. The HAE-PRO and HAE-AS provide the best detailed coverage of swelling symptoms by location, and the HAE-PRO provides the best coverage of non-swelling symptoms. Skin redness, rash, and eruptions are only covered by the HAE-PRO. Fatigue (including feeling tired and run down) is only covered by the HAE-PRO and the HAEA-QoL (Table 4).

Symptoms identified from the literature review and expert clinician interviews—but not assessed in the assessment tools themselves—included flu-like symptoms, fever, itching, and nausea. Additionally, paranesthesia, numbness, and tingling were also reported in both the literature and by clinicians, and discussed as important prodromal symptoms—yet not reflected in any instruments. Finally, less commonly reported symptoms (excessive sweating, temperature changes, mild bruising) are also not covered in the instruments (Table 4).

## Discussion

The most frequently reported symptoms in both the literature review and clinical interviews included fatigue, rash, and abdominal symptoms. During an attack, the most frequently reported symptoms in both the literature and clinician interviews focused on different swelling symptoms. While swelling symptoms were reported before or during an attack, non-swelling symptoms were also reported before and after attacks, as well as in between attacks. Although no specific consensus was reached about the definitions of “before, during, after, and between” attacks, it was generally recognized that symptoms occur outside the swelling episodes. The information gained from the literature and clinician interviews shows a much broader patient experience with HAE symptoms and impacts. Additionally, all clinicians recognized that the current focus of patient care has moved from attack-specific to a broader, more holistic definition of disease burden. Clinicians also felt that they have been hearing this broader picture of symptom-related impacts from their patients.

The current PRO tools may not fully reflect the symptom experience of HAE patients across this continuum of symptoms in their everyday lives. While the identified instruments have different recall periods, none of them assess the timeframe in which a particular symptom occurred, whether that be before, during, after, or in between an attack. More research may be needed to better define and distinctively outline these timeframes. Additional research is needed to better understand and describe the changes occurring in symptom frequency and severity, attack frequency and severity, and duration for patients living with HAE and starting new prophylactic therapies. Qualitative interviews with patients who live with HAE and have experienced different treatment regimens will be required to better understand the impact of long-term prophylactic therapy and confirm whether being attack-free for longer durations effects on patients’ emotional well-being and functional status across all time periods. Further, it is unclear whether the patient-reported HAE symptoms in this study were side effects of the prophylactic treatments being taken. Larger quantitative studies are also needed to differentiate between symptoms and treatment side effects at different timepoints.

Clinicians described their HAE patients as having difficulties with anxiety and depression, both before and after acute attacks. This suggests that patients living with HAE experience both physical symptoms and emotional impacts. Further research is needed to characterize patients’ emotional impacts, and if they change following the start of long-term prophylaxis. It is noteworthy that patients with long-term prophylactic therapy still experience prodromal symptoms that do not necessarily lead to an intensification of the swelling episode, because their anxiety level about an impending attack will likely lessen (since the usual warning signs no longer indicate such an imminent attack).

Several PRO instruments exist, and collectively they address many of the HAE symptoms faced by patients. However, of the seven PROs assessed, only the HAE-PRO adequately addressed the symptoms raised in the literature review and clinical expert interviews; however, several key areas remain unaddressed. Only the HAE-PRO assessed the attack severity and symptom duration, and only the AAS addresses the overall severity of swelling and pain—but in general, rather than for specific symptom locations. The AECT also asks participants to describe how bothersome these symptoms were and how well they were controlled by treatment. The AE-QoL and HAEA-QoL provided the most content coverage for the impacts raised by patients living with HAE, both in the literature and from clinical expert interviews. In looking at an evolving patient experience, it is important to re-evaluate what concepts are being assessed, the length of time these concepts occur, whether these are the best concepts to reflect the treatment goals, and whether they are being covered by existing HAE-specific PRO assessment tools.

## Conclusions

Although the current research is based on a small sample of expert clinicians and a targeted literature review, the results show that HAE symptoms are not limited to only those experienced during HAE swelling attacks. Many different symptoms (including abdominal symptoms, pain, headache, fatigue, depression, and anxiety) may also occur at different times. As the symptom experience varies widely across patients, clinicians have heard patients describe changes in their symptom severity and noticed a pattern of symptom occurrence from the new prophylactic treatments.

From the review conducted, very few HAE assessment tools reflect the changing patient experience and include the symptom experience in all time periods (apart from the swelling episodes). Patients experience a wide variety of HAE symptoms, thus making an assessment of all of symptoms infeasible. However, some frequently experienced symptoms, and those often preceding attacks (paresthesias, itch, nausea), should be considered as part of a thorough assessment of patient HAE status utilizing HAE PROs. Yet no existing HAE instruments reflect these concepts.

Other aspects of the symptom experience may be more important to capture, such as specific symptom severity and duration, and overall attack severity and duration—especially since these are aspects where patients and clinicians report change due to the new prophylactic treatments. These are areas that may be more meaningful to patients, and possibly more reflective of treatment benefit. With the advent of new HAE prophylactic therapies, patients experience fewer HAE attacks, and the intervals between HAE attacks may increase [13]. These therapies may additionally impact HRQoL and emotional well-being [30]. Further research is needed to determine the effect of being attack-free for longer intervals on the full symptom spectrum, including emotional well-being and physical functioning.

## Data Availability

The datasets generated and/or analyzed during the current study are not publicly available, but are available from the corresponding author on reasonable request.
